# Acknowledgment of Reviewers

**DOI:** 10.1093/geroni/igaf001

**Published:** 2025-02-11

Scientific progress depends on the generosity of reviewers who assist editors by sharing their time and expertise in the peer review process. Michelle Putnam, MGS, PhD, FGSA, Editor-in-Chief of *Innovation in Aging,* wishes to thank the following individuals for their assistance in reviewing manuscripts during 2024.

Abaei, Elnaz

Ajrouch, Kristine

Aldwin, Carolyn

Aletta, Francesco

Anderson, Keith

Anugwom, Kenechukwu

Austin, Charles

Ayalon, Liat

Baek, Jihye

Baik, Sol

Barrett, Anne

Beach, Brian

Beach, Scott

Beizer, Judy

Belmin, Joël

Ben-Noon, Rinat

Bernstein, John

Bethell, Jennifer

Bherer, Louis

Bielsten, Therese

Binfet, John Tyler

Bishop, Christine

Bishop, Nicholas

Bolt, Eva

Bonds Johnson, Kalisha

Boon, Jeffrey

Boot, Walter

Boström, Anne-Marie

Bowblis, John

Brankaert, Rens

Bristol, Alycia

Brown, Lauren

Bünning, Mareike

Burgdorf, Julia

Burnes, David

Burns, Shane

Buta, Brian

Cahill, Kevin

Cai, He

Camacho, David

Campbell, Anthony

Canning, Shelley

Cantu, Phillip

Cao, Jiawei

Carr, Deborah

Castaldo, Anna

Chan, Andrew

Chan, Thomas

Chana, Sofia Mildrum

Chatterjee, Sudeshna

Chen, Shenglan

Chen, Yu

Chisholm, Latarsha

Choi, Namkee

Choi, Yeon Jin

Christoforatos, Alexandra

Clair, Catherine

Cohn-Schwartz, Ella

Connors, Michael

Costa-Font, Joan

Crittenden, Jennifer

Czaja, Sara

Dai, Miao

Davey, Adam

Davila, Heather

De Liema, Marguerite

Degenholtz, Howard

Deng, Huiwen

Dodge, Hiroko

Douglas, Joy

Doyle Greene, Kecia

Duchesneau, Emilie

Edelstein, Offer

Egbujie, Bonaventura

Erickson, Alexander J.

Esquivel, Christi

Facal, David

Fang, Fang

Farsijani, Samaneh

Festini, Sara

Fingerman, Karen

Finn, Mary

Fishman, Paul

Flatt, Jason

Folbre, Nancy

Forrester, Sarah

Fortinsky, Richard

Frase, Robert

Froberg, Ruthann

Funayama, Michitaka

Gately, Megan

Ge, Shaoqing

Ghimire, Saruna

Gibson, Allison

Gilligan, Megan

Gladin, Amy

Glicksman, Allen

Glynn, Nancy

Goldman, Alyssa

Gomes Gonçalves, Natalia

Gubernskaya, Zoya

Guo, Xingzhi

Haghighi, Paniz

Hall, Latoya

Hall, Rasheeda

Halvorsen, Cal

Hammersmith, Anna

Han, Gang

Han, Lefei

Hanes, Douglas

Harrington, Erin

Heggenberger, Anna Lina

Hoell, Andreas

Holmes, Sarah

Hu, Yuanlong

Hua, Cassandra

Hubner, Sarah

Hughes, Jaime

Hung, Lillian

Huo, Meng

Hwang, Inuk

Idler, Ellen

Ie, Kenya

Ihara, Emily

Ikenoue, Tatsuyoshi

Ikeuchi, Tomoko

Jacobson, Mireille

Jao, Ying-Ling

Jayakody, Dona

Jiang, Da

Jilla, Anna

Joo, Jin hui

Kaplan, Daniel

Karlin, Nancy

Kaufmann, Christopher

Kim, Eunbea

Kim, Seoyoun

Kindratt, Tiffany

Kobayashi, Kimi

Koh, Vanessa

Kono, Ayumi

Kornadt, Anna

Lai, Wenhua

Latham-Mintus, Kenzie

Lee, Jeong Eun

Lee, Shaun

Lee, Soohyoung

Lee, Yeonjung

Leggett, Amanda

Leonard, Natalie

Li, Suyun

Li, Xiaojuan

Li, Yichao

Liang, Jiaming

Lin, Liang-Yuan

Lin, Yi-Yuan

Lindeman, Katja

Liu, Darren

Liu, Hui

Liu, Jingwen

Liu, Xinran

Liu, Yin

Liu, Yujun

Lloyd-Sherlock, Peter

Lon, Sou Ka

López-Ortega, Mariana

Loughnane, Megan

Lyons, Karen

Mackiewicz, Marissa

Mahmut, Mehmet

Mair, Christine

Marques, Mario

Martin, Catherine

Marx, Katherine

Matthews, Judith

Mattos, Meghan

Mbanefo, Onyinye

McConnell, Eleanor

McDermott, Cara

McInnes, Lynn

McLaughlin, Anne

Mehta, Jignasa

Meijer, Erik

Meyer, Kylie

Miller, Katherine

Miller, Lyndsey

Mitchell, Kimberly

Mitchell, Lauren

Miyawaki, Christina

Monteiro, Andrea

Montepare, Joann

Moon, Heehyul

Moored, Kyle

Morris, Ashley

Motter, Jeffrey

Muller, Tobias

Muramatsu, Naoko

Nah, Suyoung

Nguyen, Christopher

Nguyen, Tran

Ning, Jing

Nishimura, Yoshinori

Nkwata, Allan

Nudell-Cook, Elijah

O’Grady, Megan

O’Brien, Marita

Orlofsky, Sarit

Owsley, Cynthia

Pardo, Nicole

Parker, Lauren

Peeters, Geeske

Peng, Tao-Chun

Peng, Xinyuan

Penhale, Bridget

Perera, Subashan

Peterson, Colleen

Pimentel, Camilla

Pini, Lorenzo

Polk, Deborah

Pope, Thaddeus

Predovan, David

Price, Elvin

Pruchno, Rachel

Puga, Frank

Qi, Xiang

Rantanen, Taina

Reckrey, Jennifer

Redfern, Mark S.

Reichert, Monika

Rivera-Hernandez, Maricruz

Roberto, Karen

Robison, Julie

Rosso, Andrea

Sabiniewicz, Agnieszka

Sanderson, Sherry

Sands, Laura

Savla, Jyoti

Schaefer, Sabine

Schaeffer, Leann

Scharmann, Leonie

Scott, Theresa

Seo, Jeongone

Seshadri, Sandhya

Shakya, Shamatree

Sher, Chloe

Shi, Sandra

Shiovitz-Ezra, Sharon

Silverstein, Nina

Simonsick, Eleanor

Skemp, Lisa

Snow, Kimberly

Sol, Ketlyne

Song, Apei

Sou, Ka Lon

Springer, Sydney

Stites, Shana

Suanet, Bianca

Suitor, J. Jill

Suzuki, Rie

Tang, Jennifer

Tang, Shangfeng

Taylor, Harry

Tazzeo, Clare

Teaster, Pamela

Thierry, Amy

Thomson, W. Murray

Topping, Michael

Toto, Pamela

Tramujas Vasconcellos Neumann, Lycia

Tsai, Kun-Ling

Tseng, Chien-Yu “Irene”

Turner, Jennifer

Tursinai, Aviad

Van Den Bossche, Maarten

Vazquez, Eve

Venditti, Elizabeth

Vickerstaff, Sarah

Vittorio, Torri

Vordenberg, Sarah

Vu, Tai Anh

Vuolo, Michael

Wallhagen, Margaret I.

Wang, Hanqian

Wang, Kaipeng

Wang, Shanshan

Wang, Zhe

Weissberger, Gali

Wethington, Elaine

Williams, Joy

Winett, Richard

Wu, Chenkai

Wyatt, Christopher

Wyman, Mary

Xia, Xin

Xu, Jiayuan

Yan, Huang-Ting

Yang, Yi

Yao, Xiu-Qing

Yao, Yao

Ye, Minzhi

Yen, Chia-Ming

Yorgason, Jeremy

Yow, W. Quin

Zaheed, Afsara

Zajacova, Anna

Zaninotto, Paola

Zhang, Bella

Zhang, Shenghao

Zhang, Yuanbo

Zhou, Zexi

Zisberg, Anna

Zwick, Thomas

